# Recent Advances in the Optimization of Anti-TNF Treatment in Patients with Inflammatory Bowel Disease

**DOI:** 10.3390/jcm12072452

**Published:** 2023-03-23

**Authors:** Eleni Orfanoudaki, Kalliopi Foteinogiannopoulou, Eirini Theodoraki, Ioannis E. Koutroubakis

**Affiliations:** Department of Gastroenterology, University Hospital of Heraklion, Medical School, University of Crete, 71003 Heraklion, Greece

**Keywords:** biologics, Crohn’s disease, immunomodulators, therapeutic drug monitoring, ulcerative colitis

## Abstract

Despite the evolution in inflammatory bowel disease (IBD) management during the last 20 years owing to the advent of new advanced therapies, anti-TNF agents still remain the cornerstone of therapy for both Crohn’s disease and ulcerative colitis. However, this does not only secure favorable outcomes for patients considering the progressive disease character and the high likelihood of primary or secondary loss of response. Therefore, trying to reach a better treatment approach and maximize the benefits anti-TNF agents offer, optimization strategies should be examined. It has been indicated that optimizing treatment with anti-TNF enhances drug efficacy and has been associated with improved disease outcomes and a complication-free disease course. From this perspective, we aim to provide an overview of currently available data and recent advances in the practices of anti-TNF treatment optimization. Special focus has been given to the role of therapeutic drug monitoring (TDM), as well as the utility of combining anti-TNF with an immunomodulator and the treat-to-target approach.

## 1. Introduction

The introduction of anti-TNFs (tumor necrosis factors) in the therapeutic algorithm of inflammatory bowel diseases (IBD) in the late 1990s has revolutionized patient management [1]. Since then, several other promising advanced therapies, including other biologics and small molecules targeting different inflammatory pathways, have gained approval for IBD treatment and many more emerging therapies are still under investigation [2]. Despite the advances in treatment, recent network meta-analysis, in the absence of head-to-head comparative studies, still suggest that anti-TNFs are a cornerstone of therapy for both moderate-to-severe Crohn’s disease (CD) and ulcerative colitis (UC) [3,4].

As treatment goals evolve, initiating anti-TNF IBD specialists aim not only for a clinical response, steroid free remission and endoscopic healing, but also for more strict endpoints, including deep remission (histologic and transmural healing) and, somehow, modification of the natural course of the disease [5]. For some patients these are realistic and feasible targets to achieve, but as with all advanced therapies, anti-TNFs reach a far from perfect plateau as regards their efficacy (60–70%), meaning that there is still a proportion of patients who either initially lack (refractory to treatment) or in the long-term lose response [6,7]. This variability in responses is attributed to differences in pharmacokinetics and pharmacodynamics, and is defined as primary non-response and secondary loss of response, respectively. Additionally, there is also a considerable percentage of patients who will develop adverse reactions or an intolerance necessitating treatment modification or discontinuation.

Given all the above, it is considered that anti-TNFs and their optimal use are still powerful weapons in the treatment of IBD. It is of crucial importance that the best choice is made for each patient, especially when there is high risk of worse outcomes and complications. It matters even more in the first-line choice for a biologic-naïve patient, since experience has shown that in subsequent agents upon a first failure the effectiveness rates decrease [8,9]. 

Apart from acting early in the disease course and aggressively with a top-down approach, once anti-TNF therapy is commenced, optimization of the therapy should be strongly considered. The importance of such a practice is becoming increasingly acknowledged, as it is a way to enhance drug efficacy, improve treatment persistence, reduce side effects, manage costs and, therefore, improve outcomes. 

In this review, we provide current data on strategies to optimize anti-TNF therapy with a focus on therapeutic drug monitoring (TDM), along with the role of combination therapy of anti-TNF with immunomodulators and the tight control approach.

## 2. Therapeutic Drug Monitoring (TDM)

### 2.1. Anti-TNF Trough Concentrations and Antibodies

Measuring trough concentrations (TCs) and targeting a concentration within a therapeutic range has been implemented in several medications other than anti-TNFs. It is a way to avoid under- and overtreatment that could lead to impaired efficacy and safety issues, respectively. As for anti-TNFs, there are plenty of studies on the value of TC measurement, primarily for infliximab (IFX), to a lesser extent for adalimumab (ADA), and scarcely for golimumab and certolizumab pegol. This may be attributed to the former’s broader use, availability, indications, effectiveness and structure (chimeric antibody), rendering it more susceptible to immunogenicity issues.

The whole practice is based on the theory of the dose–response relationship [10]. Increased, within predefined therapeutic ranges, drug concentrations are associated with positive disease outcomes. Notably, there is a positive correlation between higher TCs and the clinical and/or endoscopic response and remission, mucοsal healing, lower relapse rates, fewer complications, fewer hospitalizations and fewer disease-related surgeries [11,12,13,14,15,16]. Moreover, optimal TCs are associated with a remarkable improvement in the patients’ quality of life, which is one of the long-term targets set out in the recent STRIDE (selecting therapeutic targets in inflammatory bowel disease) consensus [17]. 

Correspondingly, suboptimal TCs correlate with unfavorable results. In a large prospective observational study [PANTS (personalized anti-TNF therapy in Crohn’s disease (CD))], which included 1610 patients under both IFX and ADA treatment, low week (w) 14 TCs were independently associated with primary treatment failure (w14), and were highly predictive of w54 non-remission and immunogenicity [18]. This association of serum TCs with therapeutic outcomes has been confirmed in both induction and maintenance periods. As a matter of fact, achieving optimal induction or post-induction concentrations might be of most importance, due to the high inflammatory burden that has to be regulated and these concentrations may prejudge the agents’ survival thereafter. However, not all patients will show the same response to an administered anti-TNF, even with IFX whose dosage is determined by the patient’s weight. There are certain factors affecting the pharmacokinetics (PK) of anti-TNFs, meaning the drugs’ absorption, metabolism, distribution into the tissues and, lastly, its elimination from the body [10]. The key determinants of anti-TNF PK, associated with increased drug clearance, lower drug concentrations and, therefore, worse outcomes, are displayed in Table 1. Sometimes, despite even achieving an adequate drug concentration, the response may still not be satisfactory indicating a possible non-TNF driven inflammatory pathway.

Immunogenicity, characterized by the formation of anti-drug antibodies (Abs), constitutes one of the main causes of loss of response (LOR) due to an anti-TNF agent interfering with the PKs [10]. Of note, patients with positive Abs were found to run a 3-fold higher risk of LOR (relative risk = 3.2; 95% CI, 1.9–5.5; *p* < 0.0001) [21], the majority of which are formed as early as the first 12 months of anti-TNF initiation (90%) [22]. 

However, not all types of Abs impact on drug clearance. Apart from total neutralizing Abs that bind to the drug rendering it ineffective, there are also reports about the low titer of transient Abs, which lack neutralizing potential and are, therefore, clinically insignificant [23,24]. As for the technical aspects, there is a variability in the assays used for Abs detection and quantification, but a standard methodology has not been widely adopted yet. As a consequence, there is problem when interpreting Abs titles from different assays. Comparative studies have shown that drug tolerant assays designed to detect Abs, despite the presence of a circulating drug, are more accurate and should be preferred over drug sensitive ones [24,25]. Additionally, they enable earlier identification of potential positive cases, offering time for the clinician to act and optimize the therapy preventing an unlikely event of LOR [26]. In detail, the detection of transient or low-concentration Abs can be managed by treatment optimization (dose escalation, dose interval shortening and/or the addition of an immunomodulator), whereas usually the presence of higher titer Abs can lead, inevitably, to undetectable or low drug concentrations, infusion reactions and treatment failure [16].

### 2.2. Therapeutic Drug Monitoring

Based on the significant evidence in favor of the value of TCs of anti-TNFs in IBD management, the therapeutic drug monitoring (TDM) approach has gained attention during the last decade in clinical practice. According to this practice, TCs and Abs are measured and treatment is adjusted with the aim of reaching the TCs within a therapeutic range [16]. Evidently, it is a way to maintain an adequate dose and, therefore, enhance drug efficacy, prolong response durability and avoid complications. It is a valuable tool for treatment optimization, used widely for anti-TNF agents. 

Acknowledging that TCs are positively correlated with clinical outcomes, it is necessary to determine the appropriate cut-off levels to apply in TDM algorithms. However, the optimal TC threshold concentrations to achieve the maximal efficacy of anti-TNFs cannot be clearly predefined. Recent data extracted from retrospective observational reports or post hoc analyses were found to be heterogeneous for the patient sample, outcome and assay used.

From the interpretation of all the available studies in this field, it has become clear that target TCs may differ depending on the administered anti-TNF agent, the measurement time point, the IBD phenotype and severity and, lastly, but foremost, the desired outcome of interest. Regarding the time point, TCs differ as expected between the induction, post-induction or maintenance periods, with the former requiring higher concentrations justified by the higher inflammatory burden at initiation and, consequently, the higher anti-TNF fecal loses and serum needs [27]. Likewise, in the same sense, higher TCs are required for more aggressive disease phenotypes (for example penetrating or perianal CD, acute severe ulcerative colitis) and when more stringent endpoints are being considered (endoscopic or histologic remission) [27]. 

Table 2 shows a summary of the most relevant and representative studies regarding TCs in the maintenance phase and their association with different outcomes. A maintenance phase target TC of ≥3–7 μg/mL for IFX and ≥5–8 μg/mL for ADA have been suggested in order to achieve favorable outcomes [28].

Undeniably, determining the best thresholds for anti-TNF agents is not an “one size fits all” situation. When awaiting prospective data, a more personalized approach should be followed since certain populations may benefit from higher concentrations and in these cases optimal management should be offered.

To date there are two different approaches to the application of TDM, the reactive and proactive approach, respectively. Reactive TDM is performed in the setting of loss of response in a patient at induction or maintenance treatment with an anti-TNF agent, basically aiming to guide the next steps in the management, whereas proactive TDM is used prophylactically in asymptomatic patients at certain intervals and situations, so as to prevent a disease flare [27]. There is evidence that both proactive and reactive TDM, when added to routine investigations, altered the management decisions in almost half of the adult CD patients receiving anti-TNF therapy [29]. The implementation of TDM is supported by many IBD experts and societies. In official guidelines it stands more as a suggestion or a low-quality evidence recommendation owing to the conflicting results in the current literature and the absence of prospective studies and RCTs (randomized controlled trials) [30,31,32,33].

**Table 2:** Summary of most relevant and representative studies regarding TCs in the maintenance phase and their association with different outcomes.

Author, Year [Ref.]	Disease Type	TCs (mg/mL)	Outcome	Assay
IFX				
Roblin, 2017 [34]	CD	>2.1	Clinical remission	ELISA
Vande Casteele, 2015 [35]	CD	>2.8	Normal CRP (≤5 mg/L)	HMSA
Ward, 2017 [36]	CD	>3.4	Normal CRP (≤5 mg/L)	ELISA
Ward, 2017 [36]	CD	>5.7	Normal FC (<59 μg/g)	ELISA
Papamichael, 2018 [11]	CD	≥9.7	Endoscopic remission	ELISA/HMSA
Papamichael, 2018 [11]	CD	≥9.8	Histologic remission	ELISA/HMSA
Yarur, 2017 [37]	CD	>10.1	Mucosal healing	HMSA
Yarur, 2017 [37]	CD	>10.1	Fistula healing	HMSA
Adedokun, 2014 [38]	UC	>2.4	Clinical response w54	ELISA
Margo, 2017 [39]	UC	>3	Normal FC (<250 μg/g)	ELISA
Papamichael, 2017 [12]	UC	≥7.5	Endoscopic healing	HMSA/ELISA
Papamichael, 2017 [12]	UC	≥10.5	Histologic healing	HMSA/ELISA
ADA				
Nakase, 2017 [40]	CD	>5	Clinical remission	ELISA
Mazor, 2014 [41]	CD	>5.9	Normal CRP (≤5 mg/L)	ELISA
Morita, 2016 [42]	CD	>7.9	Mucosal healing	ELISA
Juncadella, 2018 [43]	CD	≥12	Endoscopic remission	HMSA
Juncadella, 2018 [43]	CD	≥12.2	Histologic remission	HMSA
Paul, 2014 [44]	CD/UC	>4.8	Clinical remission	ELISA/Radioimmunoassay
Ungar, 2016 [45]	CD/UC	>6.6	Normal CRP (≤5 mg/L)	ELISA
Yarur, 2016 [46]	UC/CD	>7.5	Endoscopic healing	HMSA
Yarur, 2016 [46]	UC/CD	>7.8	Histologic healing	HMSA
Morita, 2016 [47]	UC	>10.3	Mucosal healing	ELISA

ELISA: enzyme-linked immunosorbent assay; HMSA: homogenous mobility shift assay; CD: Crohn’s disease; UC: ulcerative colitis; IFX: infliximab; ADA: adalimumab; FC: fecal calprotectin; CRP: C-reactive protein; TCs: trough concentrations.

#### 2.2.1. Reactive TDM vs. Empiric Use of Anti-TNF

The utility of reactive TDM has been investigated in several studies and is more established in contrast to that of proactive TDM. In the setting of LOR, assessing TCs and Abs helps to identify the possible cause and manage it in a more efficient and documented way. It can be precisely identified whether failure is attributed to pharmacokinetic (immunogenic or not) or mechanistic reasons, which makes it more likely that successful management decisions are taken (optimize the current anti-TNF or change the medication). On the other hand, an empiric approach alone has been proved to be suboptimal, costly and may put the patient in jeopardy.

Reactive TDM-based treatment was found to be significantly superior in terms of clinical response, endoscopic remission, cost savings and hospitalization rates, than empiric treatment of IBD patients under IFX [48]. Regarding ADA, Restellini et al. reported that reactive TDM-guided dosing in CD patients with LOR had better outcomes than empiric dose escalation [49].

On the contrary, the GETAID (Groupe d’Etude Therapeutique des Affections Inflammatoires du Tube Digestif) team, following symptom-based treatment optimization in IBD patients under maintenance with IFX, showed similar rates of clinical, endoscopic and steroid-free remission at week 54, as the biomarker and TC-driven-based therapy [50]. Steenholdt et al., in their study, also verified the lack of superiority in clinical outcomes between conventional and reactive management, despite the significant advantage in costs in favor of the latter [51].

A systematic review and a meta-analysis of the literature concluded that reactive TDM is not better in terms of clinical efficacy versus the standard of care, however it offers a cost advantage. The authors highlighted that the data are insufficient and mostly of low quality to evidently support, for or against, this approach [15,52].

In practice, reactive TDM, if available, should be considered along with clinical evaluation, biomarkers and endoscopy to optimize treatment in the advent of LOR, gaining in costs, time and agents. In case of immunogenicity with high Ab concentrations, by following the suggested reactive TDM algorithm, time is not wasted waiting for improvement after the empirically applied dose escalation. Moreover, an agent is not easily abandoned before being completely sure that the optimal concentrations have been reached, getting the best of it. 

#### 2.2.2. Proactive TDM versus Empiric Use of Anti-TNF

Proactive TDM is defined as a periodically performed measurement of TCs and ADAs in patients in clinical remission, followed by an appropriate adjustment in the drug dosing or timing intervals with the aim of reaching certain concentrations associated with better outcomes. Apart from efficacy reasons, it has been suggested as a method to promote safety and cost effectiveness. However, the benefits of such a practice when compared to standard practice have not been convincingly proven. Maybe, the implementation of proactive TDM during or shortly after induction is theoretically of more principal importance. High inflammatory burden, usually present during induction, may cause low drug concentrations. Low week 14 TCs, both for IFX and ADA, were independently associated with primary non-response and a lack of remission at week 54 [18]. Moreover, low TCs are predisposed to antibody formation and further drug clearance, rendering it ineffective (secondary LOR); a condition not easily reversed. Retrospective analysis of the ACCENT 1 (a Crohn’s disease clinical trial evaluating infliximab in a new long-term treatment regimen) trial has also demonstrated durable sustained response through to week 54 in CD patients under IFX, achieving both satisfactory TCs at week 14 (≥3.5 µg/m) and a ≥60% decrease in CRP (C-reactive protein) [53]. Furthermore, in perianal CD IFX TCs > 9.2 μg/mL at week 2 of IFX induction were associated with fistula closure at week 14 and 30 [54]. Similar short- and long-term beneficial outcomes have been observed in studies concerning both disease types and all anti-TNF agents with variable TC limits, according to the week tested and targeted outcome (Table 3). 

An example, underscoring the value of proactive TDM during induction is that of the recognized need for accelerated IFX dosing during induction in severe acute ulcerative colitis. It was performed empirically at first, based on the understanding of underlying pharmacokinetic mechanisms influencing drug clearance [71], but eventually accelerated IFX therapy was deemed to be associated with reduced colectomy rates and less LOR episodes. Boosting effective serum concentrations during induction has proved to be beneficial in other at-risk patients, apart from those with high disease activity, like obese patients or smokers. One can guess how advantageous it could be if the accelerated dosing was performed more precisely using proactive TDM, quantifying exactly the needs caused by this inevitable medicine loss. To sum up, the TDM practice instituted early after treatment initiation could help avoid the 30% of described cases or primary non-response, and actually limit its causes to just mechanistic failure and predict better outcomes in the long term [72].

On the contrary, in the recently published Norwegian NOR-DRUM (NORwegian DRUg Monitoring) study, an RCT designed to assess the value of proactive TDM during the induction of IFX in patients with various chronic immune mediated inflammatory diseases, including IBD, found no benefit in the use of TDM when compared with standard care for clinical remission rates over week 30 [73]. 

The maintenance phase data on proactive TDM remain conflicting. TAXIT (trough level adapted infliximab treatment) and TAILORIX (a study investigating tailored treatment with infliximab in active Crohn’s disease), two out of the limited existing RCTs in this field, failed to demonstrate a significant benefit of proactive TDM in achieving remission at year one. However, fewer flares were noted in the TDM group implying a more efficient use of the anti-TNF [50,74]. On the other side, the maintenance sub-study of the aforementioned NOR-DRUM study did find significant superiority in the proactive vs. the standard approach in sustaining disease control without worsening, during the 52 week study period [75]. This was confirmed by the pediatric PAILOT (pediatric Crohn’s disease adalimumab level-based optimization treatment) study regarding ADA in CD and 1-year steroid-free clinical remission, as well as more composite outcomes (steroid-free clinical remission + normal CRP + normal FC (fecal calprotectin)) [76], and became further validated when applied for a longer time period, far from one year. Indicatively, in a 3-year Spanish prospective trial during a median follow-up at 84 [34,35,36,37,38,39,40,41,42,43,44,45,46,47,48,49,50,51,52,53,54,55,56,57,58,59,60,61,62,63,64,65,66,67,68,69,70,71,72,73,74,75,76,77,78,79,80,81,82,83,84,85,86,87,88,89,90,91,92,93,94,95,96,97,98,99,100,101,102,103,104,105,106,107,108,109,110,111,112,113,114,115,116,117,118] weeks, longer treatment durability, less disease-related surgeries or hospital admissions and less serious infusion or other adverse reactions, were noted in the proactive TDM group [77]. Moreover, even stronger target outcomes, like mucosal healing, were accomplished in higher rates than with standard management [78].

Similarly, conflicting results on proactive TDM during maintenance are displayed in retrospective observational studies. TDM-driven anti-TNF treatment (IFX or ADA) demonstrated favorable outcomes in terms of efficacy, health care utilization, surgeries, drug survival and durable drug effect not only in the short term, but also in the long term, regarding both disease types [79,80,81,82,83]. Even if applied at least once during the maintenance in patients receiving ADA, it was found to be associated with less 3-year treatment failure [83]. On the other hand, Bernardo et al. failed to show any difference between proactive TDM and the empiric arm for clinical remission, relapse and surgery [84], although time to relapse was found to be notably longer in the former. In the 3-year follow-up of the TAXIT trial patients that were assigned to either arm during the study (TDM or clinical driven dosing), the same rates of mucosal healing and treatment persistence were found [74]. However, both arms were initially optimized using the proactive TDM algorithm before randomization, which might have influenced the results. 

Two meta-analyses by Ricciuoto et al. and Nguyen et al. did not identify a significant difference in the clinical remission rates using proactive TDM versus the normal standard of care, although its implementation might provide potential advantages to the length of anti-TNF survival/persistence [15,85]. However, the authors conclude that a possible positive effect in patients with certain disease characteristics (high risk of worse outcomes) or at a certain phase (ex. induction) cannot be ruled out (possible underrepresentation at RCTs). On the contrary, Sethi et al., including both RCTs and observational studies in their meta-analysis, did find a significant association of proactive TDM with reduced treatment failure and surgical rates, as well as superiority regarding endoscopic remission. Considering all the available data and before they become further validated and substantiated, the individualized use of proactive TDM in certain cases could be recommended.

#### 2.2.3. Other Applications of Proactive TDM

The utility of proactive TDM is not restricted to exclusively guiding the treatment aimed at preventing LOR, but it has also displayed value in other clinical scenarios.

One of these scenarios has to do with treatment de-escalation of anti-TNFs for safety and cost issues when the disease is well controlled. This could be conducted either by decreasing the dose or by lengthening the time interval. TDM implementation in such a case helps to better determine the patients that can be safely and successfully de-escalated, rather than using clinical and/or biochemical parameters alone. Amiot et al., based on its association with relapse-free survival, suggested that TDM should be used prior to de-escalation in preference to symptoms and CRP [87]. Additionally, using IFX-TCs > 7 mg/L as a trough cut-off to perform de-escalation, patients were found to run a decreased relapse risk (HR: 0.45, *p* = 0.024) [88], whereas for ADA the cut-off value for successful dose reduction was 12.2 μg/mL, as described in an observational prospective study by Peris et al. [89]. Even after deintensification, continuing TDM can be beneficial for the follow-up [90]. Maintaining IFX–TCs > 2.4 μg/mL, thereafter, is critical according to Pettitcolin et al. since lower concentrations turned out to be predictive of relapse (*p* = 0.0001) [90].

Similarly, TDM could be considered to guide immunosuppressants (IMM) cessation in patients receiving combination treatments. Drobne et al. demonstrated that withdrawing IMM after at least 6 months of combination treatment did not significantly affect TCs [91]. Detectable > 0.3 μg/mL TCs at IMM cessation were associated with long-term response. Interestingly, in the same study, none of the patients with IFX–TCs > 5 μg/mL at the time of IMM withdrawal lost response during the 29-month (IQR, 15–45) follow-up period.

In cases where combination treatment cannot be used (because of a history of severe infections, malignancies etc.), there is evidence that optimized monotherapy using proactive TDM could be used. A post hoc analysis from the SONIC (study of biologic and immunomodulator naïve patients in Crohn’s disease) trial showed that after stratifying patients into quartiles based on IFX–TCs, outcomes at w26 within a quartile were comparable irrespective of IMM use [92]. Likewise, no difference in either the efficacy or drug persistence was noted when proactively optimized IFX monotherapy and combination therapy were compared [93,94]. However, the former was associated with increased drug consumption [93].

Another scenario fitting for proactive TDM, is when considering resuming an anti-TNF agent after a drug holiday. Applied early after re-exposure (before second dose), proactive TDM may help in predicting the outcomes. At that time, positive Abs predispose to severe infusion reactions and, apparently, there is a need for treatment discontinuation, whereas high TCs are related to the long-term response [95].

Lastly, and not well-established, is the role of proactive TDM peri-operatively. In the era of advanced therapies most patients at the time of surgery have been exposed or are currently being exposed to biologic treatment. Lau et al. reported a positive relationship between TCs and adverse post-operative outcomes in CD patients [96]. Unlike, in the PUCCINI (prospective cohort of ulcerative colitis and Crohn’s disease patients undergoing surgery to identify risk factors for post-operative infection) trial, the biggest prospective observational study in this field, which included 947 patients undergoing surgery (382 patients were exposed to anti-TNF within 12 weeks of surgery), no association was found between detectable TCs and post-operative infections (surgical site or not) [97]. The outcomes were comparable in smaller cohort studies, demonstrating no difference in post-operative complications irrespective of anti-TNF exposure, the titer of TCs, or the time interval from surgery to the last dose [98,99]. The current practice is not to delay surgery for reasons like recent anti-TNF exposure. 

### 2.3. Limitations and Challenges Implementing TDM

Based on the current evidence, TDM could not be recommended for wide use in clinical practice. Although the additional guidance it offers in the management of IBD is quite established, there are several aspects that have not been fully clarified. 

There are still knowledge gaps regarding the optimal application of TDM, result interpretation and determination of the optimal thresholds to target. This can be attributed partially to the lack of standardization, the heterogeneity in populations and outcomes across most studies, and the high variability of testing kits and assays, especially for antibody detection, causing confusion in result interpretation. 

There is also an evident difficulty in obtaining the results in a timely fashion, so as to drive early therapy decisions. The commonly used ELISA (enzyme-linked immunosorbent assay), has a turnaround time of 2–4 weeks causing a delay in the implementation of changes. Moreover, most experience refers to IFX rather than other anti-TNF agents, mostly the maintenance phase rather than the induction and, last but not least, the cost issue owing to the lack of reimbursement from insurance companies. 

Another issue that has yet to be addressed, is that of the best timing within a treatment circle for the serum concentrations to be measured. Conventionally, serum anti-TNF concentrations are measured at the trough, meaning at their expected lowest point just before the scheduled re-administration. However, better understanding of the underlying pharmacokinetics has led to the assessment of concentrations other than at the trough, like at the peak or intermediate point. There is emerging evidence on the utility and value of these concentrations in predicting remission, particularly if implemented at the induction [100]. This is usually the case for IFX, since for ADA concentrations measured any time in between a single treatment circle seem to be comparable (POETIC study (prospective observational evaluation of time-dependency of adalimumab immunogenicity and drug concentration)) [65]. In this context, tissue drug concentrations are also being considered and their concentrations in noninflamed tissue are associated with higher sustained response and remission [101].

### 2.4. Current Perspectives 

In order to overcome the time lag between TCs measurement and result availability, point of care (POC) methods have been developed for the evaluation of TCs and Abs. They are rapid assays carried out at the point where care is provided, making accurate results feasible within minutes. Regarding TCs measurement, POC assays exhibit high correlation with standard ELISA, with an overall agreement of up to 89.4% [102,103]. Moreover, POC tests can detect Abs accurately and reliably enough, with 100% specificity and 76% sensitivity, and their results are highly correlated with those from the ELISA assays too [104]. Despite the proven non-inferiority of POC tests, a revision of the currently suggested TC thresholds should be considered, as in POC tests TC values seem to be higher [105]. In clinical practice ultra-proactive TDM applied using POC tests was no different in rates of IFX failure or sustained clinical remission after 1-year when compared with a once-applied reactive TDM [106].

Another emerging attempt toward a more personalized approach, is the use of dashboard-driven TDM. By entering patients’ data, including pharmacokinetic parameters like CRP and albumin that influence drug clearance, dashboards offer guidance on optimal dosing and treatment intervals by indirectly forecasting TCs. The PRECISION trial demonstrated lower LOR rates and FC values 1-year after application of dashboard-driven dosing in patients with IBD in remission under maintenance treatment with IFX in comparison with the normal standard of care [107]. If applied earlier to guide accelerated IFX induction dosing, a PK dashboard may improve drug durability and immunogenicity [108]. In the meantime, an RCT is ongoing (the OPTIMIZE trial) also focusing on the induction phase, with the aim of assessing remission rates and the need for rescue therapy from week 14 through to week 52 in patients with moderate-to-severe CD initiating IFX and comparing the proactive TDM dashboard-driven dosing with the normal standard of care [109].

The combination of these innovations, point-of-care testing and dashboard-driven TDM, introduced early at anti-TNF initiation could potentially further maximize the outcomes (drug survival, effectiveness, costs, safety).

## 3. Combination Therapy

Several studies suggest that combination therapy of an anti-TNF with an immunomodulator (thiopurine or methotrexate (MTX)) has a superior effect over anti-TNF monotherapy in IBD [110,111] (Table 4). The main mechanism explaining combination therapy superiority is the effect of the combination therapy on the immunogenicity of the anti-TNF. The risk of Abs development in patients under combination therapy seems to be lower than in those under monotherapy. The presence of Abs leads to lower serum TCs, less probabilities of therapy success and higher rates of loss of response. IFX use is more likely to develop Abs because of its structure, and this may explain the fact that most trials supporting combination therapy refer to IFX [112]. ADA antibody formation rates are lower than for IFX, however, there is still a benefit from using it in combination with an immunomodulator over monotherapy [113]. Another possible mechanism explaining the benefits of combination therapy, regarding thiopurines, is their separate ability to manage disease activity or even, mucosal healing [114,115].

### 3.1. IFX Combination Therapy

There is strong evidence, coming from large clinical trials, supporting the use of combination therapy of IFX with an immunomodulator in IBD. The SONIC trial showed that combination therapy of IFX with AZA (azathioprine) displays better outcomes compared with monotherapy [116]. Correspondingly, regarding UC, the SUCCESS study highlighted the superiority of the combination of IFX with AZA against IFX monotherapy in naïve UC patients [117].

The ECCO (European Crohn’s and Colitis Organization) recommends the combination of IFX with a thiopurine for the induction of remission in patients with moderate-to-severe Crohn’s disease after a failure in response to conventional therapy [30]. The AGA (American Gastroenterological Association), as well, suggests the use of IFX combined with a thiopurine for the induction and maintenance therapy of naïve to both biologics and thiopurine patients, and comments that the combination of IFX with methotrexate may as well be preferred over monotherapy [32].

Notably, concerning combination therapy with MTX, there is no clear evidence to support its use to improve IFX efficacy, since the data remain rather conflicting. 

### 3.2. Adalimumab Combination Therapy

In contrast to what applies for IFX, there are differences in the guidelines regarding the necessity of concomitant immunosuppression regarding treatment with ADA. The ECCO does not recommend the combination to achieve clinical response and remission [30], whereas the AGA guidelines favor the use of combination therapy with thiopurines for the induction and maintenance of remission over ADA monotherapy in naïve CD patients. Furthermore, according to the AGA recommendations, the combination therapy of ADA with MTX may be also considered [32].

The Diamond study, evaluating the utility of ADA in combination or not with a thiopurine showed no superiority of the former over monotherapy in the achievement of clinical remission in week 52. However, combination therapy was associated with higher endoscopic remission rates in week 26 [118].

Targownik et al., in a retrospective analysis of more than 70,000 patients with CD and UC, compared the use of IFX or ADA in combination with an immunosuppressant, either thiopurine or methotrexate.

Any combination therapy was found to be associated with a significant decrease in treatment failure for both disease types. ADA and IFX were equally effective (in combination with an immunomodulator) in CD, but regarding UC, thiopurines performed better over methotrexate in combination with an anti-TNF [122].

### 3.3. Anti-TNF Experienced Patients

An important issue is that most of the available data come from clinical trials with patients naïve to biologics. This seems to be only a part of the real-world management. In fact, a respectable proportion of patients previously exposed to anti-TNF need to change therapy to another anti-TNF and this turns out to be rather challenging. However, in a recent study the use of combination therapy versus monotherapy in patients with previous immunogenicity related failure, changing to another anti-TNF was evaluated. Patients previously exposed to IFX changed to either ADA or ADA combined with AZA, and patients previously exposed to ADA changed to either IFX or IFX combined with AZA, respectively. Combination therapy was the only factor associated with better outcomes at 24 months [120]. 

### 3.4. Combination Treatment with Immunomodulator: Dosage, Timing and Safety

Although the value of combination treatment has been clearly demonstrated, there are certain other issues that have not been fully clarified yet.

The recommended therapeutic thiopurine dose in IMM monotherapy is 2–2.5 mg/kg for azathioprine and 1–1.5 mg/kg for 6-MP (6-mercaptopurine). It seems that when applied as combination therapy, not as high doses are needed. In a recent study by Ramos et al., remission, response or failure rates using anti-TNF (IFX or ADA) in combination treatment with either a standard (2–2.5 mg/kg) or decreased (<2 mg/kg) azathioprine dose, were similar in the two groups. Anti-TNF (IFX or ADA) serum concentrations and Abs formation, as well as endoscopic or biochemical remission, in the two groups were similar [124,125]. Even if thiopurine was started in full dose and patients were in durable remission under combination treatment, dose reduction shows equal efficacy performance as its maintenance at full dose [126].

Considering the duration of the combination treatment, data are rather conflicting. Lambrescak et al. in a retrospective cohort study, which included a group of 139 patients with CD or UC, showed that a shorter duration of IFX combination therapy with an immunomodulator (thiopurine or MTX) was not associated with a higher risk of treatment failure. Moreover, in patients where the immunosuppressant was discontinued, a combination therapy of 6–11 months was not associated with a higher risk of treatment failure compared with those with a duration of combination therapy >12 months [127]. 

On the other hand, in patients with CD in clinical remission, a duration of combination therapy with IFX and AZA of less than 27 months was associated with higher rates of relapse over the withdrawal of AZA, after a period of 27 months [128]. Additionally, Mahmoud et al., in a retrospective cohort study, showed that the immunomodulator withdrawal was not associated with a higher risk of loss of response, but the presence of Abs was more frequent in the serum of these patients over those with uninterrupted use of combination therapy. Notably, a longer period of immunomodulator use before withdrawal and higher TCs at the time of withdrawal were associated with a lower risk of Abs formation. Furthermore, higher TCs and clinical remission at the time of withdrawal were also associated with lower risk of loss of response [123]. Apparently, there is no optimal duration for combination treatment and an individualized approach should be considered.

As for the timing of the immunomodulator administration, the simultaneous initiation of both anti-TNF and IMM is preferable. Chen et al. in a retrospective study, which included more than 9000 patients with UC and CD, showed that biologic therapy (mainly anti-TNF) in combination with an immunosuppressant is related to a lower risk of biologic discontinuation and of note, when the immunomodulator therapy was started more than 30 days before the biologic induction, the risk of discontinuation of the biologic was even lower [129]. Patients starting IMM at the time of IFX induction also displayed lower incidence rates of Abs formation, contrary to those starting concomitant IMM later [113]. At this point it is also worth mentioning that an IMM may as well be added later in the treatment with anti-TNF in case of LOR, since it plays a role not only in preventing, but also in suppressing or even eliminating antibody formation, as well as in recapturing a response [130,131]. 

Lastly, the use of a combined therapy raises concerns about safety. There is evidence that combination therapy is not associated with more adverse outcomes, but in some specific patient groups there is, nevertheless, still a need for more caution; this is the case because it has been associated with a higher risk of lymphoma and serious infections [132,133]. More specifically, young men may be at a higher risk of hepatosplenic T-cell lymphoma and the elderly may be at a higher risk of lymphoma and infections [134,135]. 

In conclusion, the combination of anti-TNF agents with an immunomodulator is one of the most efficacious treatments available. Early initiation of combination therapy is recommended as part of the top-down approach for high-risk patients (with risk factors for worse outcomes and complications, aggressive disease behavior) with moderate-to-severe disease, although the evidence regarding adalimumab is not strong enough yet. Combination therapy has also proved beneficial in anti-TNF experienced patients in the case of immunogenicity-related failure. For low-risk patients or for patients with concerns regarding safety, monotherapy is suggested, preserving the IMM addition when LOR or immunogenicity issues arise. Moreover, therapy personalization with the right benefit–risk assessment and the awareness and participation of the patient in the decision making, may lead to the best approach to optimize IBD therapy.

## 4. Treat-to-Target Approach in IBD

In IBD, like other chronic diseases, it is of high importance to set clear therapeutic targets, in order proper disease management to be adopted and major IBD complications, hospitalizations, surgeries, and poor quality of life to be avoided. According to STRIDE I, concerning IBD treatment, the alleviation of clinical symptoms or else the so-called clinical response/remission and endoscopic remission as well, were identified as the therapeutic goals, short-term and long-term targets, respectively [136]. As for the resolution of clinical symptoms, it became obvious over the years that this primary target was not enough to ensure neither the resolution of bowel inflammation nor the prevention of permanent intestinal damage leading to surgery [137]. In the aforementioned SONIC trial, 50% of CD patients who were in clinical remission, as expressed by the CDAI (Crohn’s Disease Activity Index) score, had endoscopic and/or biochemical (CRP) active disease [138]. Thus, STRIDE II updated the treat-to-target strategy, incorporating serum and fecal biomarkers as the intermediate targets in between the short- (symptomatic response) and long-term targets (endoscopic healing) [17]. 

### 4.1. Biomarkers in IBD

The most commonly used biomarkers in everyday clinical practice for IBD patients are serum C-reactive protein (CRP) and fecal calprotectin (FC). According to a meta-analysis, the pooled sensitivity and specificity of CRP and FC were 49% (95% CI 34–64%) and 92% (95% CI 72–96%), and 73% (95% CI 66–79%) and 82% (95% CI 73–88%), respectively. The FC was more sensitive than the CRP in both disease types and was more sensitive in ulcerative colitis than Crohn’s disease [139]. In another more recent meta-analysis, the FC showed a pooled sensitivity of 85%, specificity of 75%, DOR (diagnostic odds ratios) of 16.3 and AUC (area under the curve) of 0.88, for assessing endoscopically active disease [140]. Regarding the FC cut-offs, the best sensitivity (90.6%) was found at 50 μg/g and the best specificity (78.2%) at >100 μg/g. For CD patients, a FC cut-off of 250 mg/g is highly predictive of mucosal healing, whereas a stricter cut-off of 100 mg/g is suggestive of deep healing (mucosal and transmural) [17]. Likewise, in UC different FC cut-offs reflect different degrees of mucosal healing as well, 250 mg/g, 150 mg/g and 100 mg/g endoscopic improvement, mucosal healing and histologic healing, respectively [17]. It is of note that consecutive FC measurements, rather than a single one, are useful for predicting short-term clinical relapses (>261 μg/g with AUC = 0.901, sensitivity 87.2%, specificity 85.3%, *p* < 0.001) [141]. 

As mentioned before, while FC may show better sensitivity, CRP has higher specificity in identifying active intestinal disease. Low CRP values have been associated with a lower risk of clinical relapse, with AUC of 0.72 and an optimal cut-off of 1.0 mg/L, with a positive and negative predictive value of 21% and 94%, respectively [142]. Moreover, a high CRP value at the time of anti-TNF discontinuation is associated with a higher relapse risk [143], whereas normalization of CRP after treatment initiation is predictive of maintained response or remission through to w54 in CD patients under IFX [144]. In the ACCENT trial, a CRP decrease > 60% at week 14 was significantly associated with sustained response to IFX [53]. It seems that a baseline CRP and CRP reduction rate could be useful in order to predict the primary non-response or the secondary loss of response to anti-TNF in patients with CD, and could possibly act as a guide for choosing the right therapeutic strategy [145]. 

The CALM (efficacy and safety of two treatment algorithms in adults with moderate-to-severe Crohn’s disease) study for CD was the first trial to show that patients in the tight-control group (escalation of anti-TNF treatment based on clinical scores, biomarkers or prednisone use in the previous week) achieved higher rates of mucosal healing [CDEIS (Crohn’s Disease Index of Severity) < 4] at week 48 in comparison to the clinical management group (escalation of anti-TNF treatment based only on clinical scores or prednisone use in the previous week) (46% of 122 patients) over (30% of 122 patients), respectively, with an adjusted risk difference of 16·1% (95% CI 3·9–28·3; *p* = 0·010)) [146]. A subsequent analysis, which included 122 patients from the CALM study with early moderate-to-severe CD, showed that deep remission (CDEIS < 4, absence of/with no deep ulcers or steroids, for eight or more weeks) was significantly associated (aHR (adjusted hazard ration), 0.19; 95% 0.07–0.31) with a decreased risk of severe disease complications, independently of the tight control or conventional management strategy [147]. 

As far as UC is concerned, in refractory disease treated with IFX, independent predictors of colectomy-free survival were found to be mucosal healing (OR, 4.02; 95% CI, 1.16–13.97; *p* = 0.028), baseline CRP of 5 mg/L or less (OR, 2.95; 95% CI, 1.26–6.89; *p* = 0.012) and baseline albumin of 35 g/L or greater (OR, 3.03; 95% CI, 1.12–8.22; *p* = 0.029) [148]. It is well-established that in UC, FC has a better sensitivity compared to CRP in predicting the endoscopic activity of the disease [136]. 

Furthermore, FC has been established as a useful predictor of clinical relapses [149]. There is evidence showing that a combination of biomarkers and other factors, such as IFX trough concentrations and Abs to IFX at week 22, are associated with secondary loss of response to anti-TNF [150]. Similarly, a combined endpoint (CDAI < 150 and CRP ≤ 2.9 mg/L and FC improvement) in CD patients after anti-TNF induction at week 12 was found to predict corticosteroid-free remission at week 52 [151]. These results suggest that possibly a combination of clinical scores, IFX concentrations as well as serum and fecal biomarkers could be used to guide and optimize IBD treatment.

On the other hand, opposed to the value of the tight-control approach, highlighted above, there are some recent conflicting data from the STARDUST (treat-to-target versus the standard of care for Crohn’s disease patients treated with ustekinumab) trial, which suggests that implementing the standard of care strategy in patients with CD treated with ustekinumab is not inferior in terms of the endoscopic response at week 48 versus the treat-to-target one [152].

### 4.2. Future Perspectives for IBD Treatment Targets

As it is already known, inflammation spreads continuously and in a superficial manner in UC, while on the contrary, in a patchy and more transmural manner in CD. For this reason, histological remission in UC and transmural healing in CD could possibly act, in the future, as the next long-term targets after endoscopic remission and mucosal healing. According to a large meta-analysis, which included 28 studies and 2806 patients (2677 UC; 129 CD), there was evidence to suggest that patients with UC who were in endoscopic remission, faced a higher risk of relapse in case of persistent histologic activity (OR 2.41, 95% CI 1.91–3.04). On the contrary, no association was found between histologic activity and relapse in CD [153]. In the same way, according to another meta-analysis, UC patients with histologic remission faced a 63% lower risk of clinical relapse vs. those with persistent histologic activity (RR, 0.37; 95% CI, 0.24–0.56), whereas the annual risk of clinical relapse in those achieving histologic remission was only 5.0% (95% CI, 3.3–7.7) [154].

When it comes to CD, it was shown that transmural healing (defined as a bowel wall thickness of ≤3 mm at bowel sonography) was associated with a higher rate of steroid-free clinical remission (95.6%), lower rates of hospitalization (8.8%) and surgeries (0%) at 1-year versus mucosal (75%, 28.3% and 10%, respectively) and no healing (41%, 66.6% and 35.5%, respectively) (*p* < 0.001) in patients treated with anti-TNF for 2 years [155]. In another observational study, which included 214 CD patients that underwent MRE (magnetic resonance enterography) and colonoscopy every 6 months, endoscopic remission (OR 0.331 95% CI, 0.178–0.614, *p* < 0.001) and MRE remission (OR 0.270 95% CI, 0.130–0.564, *p* < 0.001) were independently associated with a lower risk of adverse outcomes (surgeries, hospitalization, bowel damage) [156]. What is more, early transmural healing as it was expressed by MRE at week 12 after anti-TNF treatment (defined as a 25% decrease in either the Clermont score (odds ratio (OR) = 7.7 (1.7–34.0), *p* < 0.001) or the Magnetic Resonance Index of Activity (OR = 4.2 (1.3–13.3), *p* = 0.015)), was shown to be associated with sustained clinical remission (corticosteroid-free remission at week 52) and prevention of long-term bowel damage in CD (HR 0.21 (0.0–0.9); *p* = 0.037) [157].

Nevertheless, currently neither the transmural healing in CD nor the histological remission in UC constitutes formal targets in the treatment algorithm of IBD. These targets have not yet been incorporated into the treat-to-target IBD strategy and further studies and RCTs are needed in order for the benefits of such a therapeutic algorithm to be proven.

## 5. Concluding Remarks

IBD management has evolved over the past two decades owing to the emergence of several biologics. Anti-TNFs were the first biologics to be approved for this indication and, notably, remain among the most efficacious agents. What is more, IFX and ADA were ranked highest as first-line treatments for the induction of clinical remission in both CD and UC patients, offering a distinct advantage over other therapies [3,158]. However, this does not always preclude an unfavorable outcome for patients, given the progressive disease character and the high likelihood of primary or secondary loss of response. Hence, the need for treatment optimization, even from the outset, has emerged in an effort to make the best use of them, preserve their effectiveness and maximize their durability.

TDM can facilitate decision making through the determination of the treatment failure mechanism. Especially when performed in a reactive setting, but also proactively in certain scenarios, TDM has proved to be beneficial in terms of recapturing a loss of response and prolonging anti-TNF durability and its advantageous outcomes. Similarly, the value of combination treatment of anti-TNF with an immunomodulator has been sufficiently established for IFX and in high-risk patients, or those with previous experience of another anti-TNF. Lastly, the achievement of a tight-disease control, by applying a treat-to-target strategy helps to accomplish improved and efficient management. Setting and reaching short-term targets, like the normalization of CRP or FC, that satisfactorily reflect the current activity enables a more complication-free disease course.

Despite the advances in all the three fields mentioned, there are still limitations and challenges that have to be overcome. Additionally, all these should be implemented in practice with a critical appraisal and an individualized approach. So far, due to the lack of strong evidence recommendations, there is substantial variability in optimization practices. Further, well-designed prospective studies and RCTs are needed to elucidate the role of TDM, combination treatment and tight control to enhance anti-TNF treatment.

## Figures and Tables

**Table 1:** Factors affecting the pharmacokinetics of anti-TNF agents [10,19,20].

Factors	Effect on TCs
Anti-drug antibodies (+)	↓
Combination therapy with immunomodulators	↑
Low albumin levels	↓
High CRP levels	↓
High BMI	↓
Male gender	↓
High serum TNF concentrations	↓
Polymorphisms in the neonatal Fc receptor	↓
Pegylation	↑

↓: decrease; ↑: increase.

**Table 3:** TDM thresholds during induction treatment and associated short- and long-term therapeutic outcomes for anti-TNF agents.

Author, Year [Ref.]	Study Design	Disease Type	Anti-TNF Agent	Patients (N)	TCs (mg/L)	Week	Outcome
Dreesen, 2020 [55]	Post hoc analysis of RCT	CD	IFX	122	>23.1 >10	2 6	Endoscopic remission week 12 Endoscopic remission week 12
Davidov, 2017 [54]	Retrospective	CD fistulizing	IFX	36	>9.3 >7.3	2 6	Fistula response week 14 Fistula response week 14
Papamichael, 2021 [56]	Post hoc analysis of RCT	CD fistulizing	IFX	282	≥20.2 ≥15 ≥7.2	2 6 14	Complete remission week 14 Complete remission week 14 Complete remission week 14 (defined as combined complete fistula response and CRP normalization)
Clarkston, 2019 [57]	Prospective	CD pediatric	IFX	72	≥26.7 ≥15.9	2 6	Clinical response week 14 Clinical response week 14
Gonzi, 2017 [58]	Prospective	CD	IFX (biosimilar)	184	>16.9 >20.4	2	Clinical response week 14 Clinical remission week 14
Gonzi, 2017 [58]	Prospective	UC	IFX (biosimilar)	107	>11.5 >15.3	2	Clinical response week 14 Clinical remission week 14
Gonzi, 2017 [58]	Prospective	UC	IFX (biosimilar)	107	>11.5 >14.5	2	Clinical response week 30 Clinical remission week 30
Kobayashi, 2016 [59]	Post hoc analysis of RCT	UC	IFX	82	>21.3	2	Clinical response week 14
Bar Yoseph, 2018 [60]	Retrospective case control	CD	IFX	140	<6.8	2	Primary non-response week 14
Vande Castelle, 2019 [61]	Post hoc analysis of RCTs	UC	IFX	484	≥18.6 >10.6 ≥5.1 ≥6.7	2 6 14 14	Mayo endoscopic score ≤1 week 8 Mayo endoscopic score ≤1 week 8 Mayo endoscopic score ≤1 week 30 Mayo endoscopic score 0 week 30
Papamichael, 2016 [62]	Retrospective	UC	IFX	101	≥28.3 ≥15 ≥2.1	2 6 14	Short-term mucosal healing weeks 10–12 (Mayo endoscopic score ≤ 1) Short-term mucosal healing weeks 10–12 (Mayo endoscopic score ≤ 1) Short-term mucosal healing weeks 10–12 (Mayo endoscopic score ≤ 1)
Adedokun, 2014 [38]	Post hoc analysis of RCTs	UC	IFX	728	≥22 >5.1	6 14	Clinical response week 8 Clinical response week 30
Kennedy, 2019 [18]	Prospective	CD	IFX	955	>7	14	Remission week 14 and 54 (CRP ≤ 3 mg/L and HBI≤ 4, no ongoing steroid therapy, and no exit due to treatment failure)
Cornille, 2014 [53]	Post hoc analysis of RCT	CD	IFX	291	≥ 3.5	14	Sustained clinical response up to week 54
Tighe, 2017 [63]	Prospective	CD/UC	IFX	17	>4.8	14	Predicts clinical response week 14
Bortlik, 2013 [64]	Retrospective	CD	IFX	84	>3	14	Sustained clinical response, decreased risk of treatment failure
Ungar, 2018 [65]	Prospective	CD	ADA	98	>6.7	2	Clinical remission week 14
Verstockt, 2018 [66]	Prospective	CD	ADA	116	>12 <8.3	4 4	Biological remission at week 12 Positive anti-drug antibodies by week 12
Vande Castelle, 2019 [67]	Prospective	CD	ADA	28	>7.3	4	Clinical remission week 12
Papamichael, 2017 [68]	Retrospective	UC	ADA	43	≥7.5	4	Mucosal healing weeks 8–14
Baert, 2014 [69]	Retrospective	UC	ADA	73	≥4.6 ≥7	4	Clinical response week 12 Clinical response week 52
Tighe, 2017 [63]	Prospective	CD/UC	ADA	18	>3.5	4	Predicts clinical response week 4
Baert, 2016 [70]	Retrospective	CD	ADA	148	<5	4	Development of anti-drug antibodies

RCT: randomized clinical trial; CD: Crohn’s disease; UC: ulcerative colitis; IFX: infliximab; ADA: adalimumab; CRP: C-reactive protein; TCs: trough concentrations.

**Table 4:** Summary of the studies using combination therapy versus monotherapy in patients with IBD.

Author, Year [Ref.]	Study Design	Patients (N)	Male (%)	Disease Type (UC/CD)	Age (Median Years)	Disease Duration (Median Years)	Follow-up (Months, Median)	Monotherapy	Combination Therapy	Concomitant Therapy	Primary Endpoint	Outcomes
Colombel, 2010 (SONIC) [116]	Randomized, double-blind trial	508	52	CD	34	2.3	11.5	IFX standard dose 5 mg/kg	IFX + AZA standard dose 2.5 mg/kg	Mesalamine, steroids	Steroid-free clinical remission at week 26	Combo better outcome vs. monotherapy (*p* = 0.02)
Panaccione, 2014 (SUCCESS) [117]	Randomized, double-blind trial	239	54	UC	38	NA	4	IFX standard dose 5 mg/kg	IFX + AZA standard dose 2.5 mg/kg	Steroids (tapering after the induction)	Steroid-free remission at week 16	Combo better outcomes vs. monotherapy ( *p*= 0.017)
Schröder, 2006 [119]	Randomized, controlled, open label, clinical trial	19	42	CD	35	9	11.2	IFX 5 mg/kg	IFX + MTX 20 mg in weeks 0–5 and then 20 mg orally weekly	5 ASA steroids (tapering)	Clinical remission (CDAI < 150)	Combo achieved remission in 91% vs. 50% in monotherapy (*p* = 0.04), earlier (2 w vs. 18 w)
Matsumoto, 2016 (Diamond trial) [118]	Multicentre, randomized, prospective, open label study	176	72	CD	31	At least 3 months	12	ADA standard dose (40 mg/2 w)	ADA standard dose + AZA 25–100 mg	5 ASA, steroids	Clinical remission (SCAI < 150) at week 26	No difference in clinical remission in the two groups (*p* = 0.63)
Roblin, 2020 [120]	Randomized, open label and prospective trial	100	49	CD/UC	39.5	3.5	24	IFX standard dose (to pts with previous failure to ADA intensified dose) ADA standard dose (to pts with previous failure to IFX intensified dose)	IFX standard dose + AZA 2.5 mg/Kg ADA standard dose + AZA 2.5 mg/kg	NA	Clinical failure and occurrence of undesirable effects at 24 months	Combo better outcome vs. monotherapy, (*p* < 0.001)
Feagan, 2014 [121]	Double-blind, placebo-controlled trial	126	56	CD	39.5	10	11.5	IFX (5 mg/kg at weeks 1, 3, 7, 14, 22, 30, 38 and 46)	IFX (5 mg/kg at weeks 1, 3, 7, 14, 22, 30, 38 and 46) + MTX (10 mg/w to 20 mg/w at week 3, and to 25 mg/w at week 5 till week 50)	Folic acid, antibiotics, steroids tapering	Time to clinical failure	No significant difference (*p* = 0.63)
Targownik, 2020 [122]	Single, open label, retrospective clinical trial	78,413	50.1	CD/UC	NA (the majority between 25–65)	1.2–8.7	NA	IFX or ADA	IFX +MTX/thiopurine, ADA + MTX/thiopurine	NA	The first occurrence of treatment failure (IBD-related hospitalization, IBD-related surgery, new/recurrent corticosteroid use or anti-TNF switch for 52 weeks)	Combo therapy was associated with a significant decrease in treatment ineffectiveness for both CD and UC (aHR 0.77 (95% CI 0.66–0.90) for CD) (0.72 (95% CI 0.62–0.84) for UC)
Mahmoud, 2022 [123]	Retrospective cohort study	543	45	CD/UC	33.5	4.1	20.4	IFX/ADA monotherapy after discontinuation of concomitant MTX/thiopurine	IFX/ADA + MTX/thiopurine	NA	LOR, detection of anti-drug antibodies to anti-TNF therapy	Immunomodulator withdrawal did not increase the risk of LOR (aHR 1.08; 95% CI, 0.72–1.61),but it was associated with an increased risk of anti-drug antibodies in the entire cohort (aHR, 2.14; 95% CI, 1.17–3.94)

MTX: methotrexate; AZA; azathioprine; CD: Crohn’s disease; UC: ulcerative colitis; IFX: infliximab; ADA: adalimumab; NA: not applicable; LOR: loss of response; TCs: trough concentrations.

## Data Availability

No new data were created or analyzed in this study. Data sharing is not applicable to this article.
